# Characterization of Fibronectin-Adherent, Non-Fibronectin-Adherent, and Explant-Derived Human Dental Pulp Stem Cell Populations

**DOI:** 10.3390/dj13040159

**Published:** 2025-04-02

**Authors:** Heoijin Kim, Shelley J. Williams, John S. Colombo

**Affiliations:** Department of Biomedical Sciences, School of Dental Medicine, University of Nevada, Las Vegas, NV 89106, USA; heoijinkimortho@gmail.com (H.K.); shelleyj.williams@unlv.edu (S.J.W.)

**Keywords:** characterization, dental pulp, DPSC markers, fibronectin selection, isolation, mesenchymal cells, osteodifferentiation, population doubling, pulp explant, stem cells

## Abstract

**Background/Objectives:** Dental pulp stem cells (DPSCs) are of significant interest due to their mesenchymal lineage and relative availability from extracted teeth. This study aims to examine the relationship between fibronectin-adherent, non-fibronectin-adherent, and explant-derived DPSC populations in terms of the population doubling rate in culture and the expression of mesenchymal cell surface markers and their capacity for osteodifferentiation. **Methods:** Human pulp tissue was removed from healthy extracted human teeth, enzymatically digested prior to seeding onto fibronectin-coated plates, and left to adhere for 20 min, yielding a fibronectin-adherent population. The remaining non-adherent cells were transferred and designated ‘non-fibronectin-adherent.’ Intact pulp was placed on uncoated plastic for 5 days, with the migrated cells designated ‘explant-derived’. DPSCs from these populations were examined in terms of population doubling rates, the expression of CD90, CD44, CD105, and CD73, and the expression of *RUNX2*, *SPP1*, and *BGLAP* after 7 days in osteoinductive media. **Results:** The fibronectin-adherent cells had the greatest population doubling over time. All populations demonstrated comparable percentages of cells positive for mesenchymal markers, though individual marker expression varied slightly. The explant-derived cells showed increased expression of *RUNX2* after 7 days in osteoinductive media, while the treated single-cell-suspension-derived populations showed increased expression of *SPP1* mRNA. **Conclusions:** Fibronectin enrichment resulted in a population with the greatest rate of population doubling over extended culture compared to the other two populations. The proportion of cells positive for all four mesenchymal surface markers was the same between populations. The fibronectin-adherent and non-adherent cultures may have responded more rapidly to osteoinductive media than the explant-derived cells.

## 1. Introduction

Dental pulp stem cells (DPSCs) are a class of multipotent mesenchymal progenitor cells that have attracted a significant amount of interest in tissue engineering and regenerative dentistry due to their neural crest origin, relative accessibility from extracted or shed human teeth, and their retention of capacity for differentiation down multiple lineages after cryopreservation [1]. It has been well established that DPSCs have the potential to differentiate into multiple connective tissue lineages and have similar regenerative properties to bone-marrow-derived Mesenchymal Stem Cells (MSCs) [2,3,4]. Fundamental challenges remain regarding translation into applications where these cells might be extracted and used within regenerative medicine. Perhaps the most significant of these is the fact that the DPSC population is relatively small, constituting around 1% of the total cells in the pulp tissue [5]. Even the most effective isolation of this small population will require significant expansion in vitro in order to achieve the number of cells required for most clinical applications. There have been wide-ranging attempts to isolate populations of DPSCs without altering their ability to proliferate or their fundamental multipotency, including clonal isolation of single cells from digested pulp tissue, flow sorting, ramen spectroscopy, and harvesting from whole pulp explants [6,7,8,9,10]. Despite the promising advances in techniques to isolate highly proliferative subpopulations of DPSCs that maintain their lineage versatility, there is still much that is not fully understood about the fundamental properties of DPSCs generated by these techniques in terms of their suitability for use in tissue engineering applications. Better optimized isolation techniques, using an initial selection with relevant biomolecules from native extracellular matrices, such as fibronectin to enhance the proliferation of isolated populations and the preservation of DPSC characteristics, may help to address these current challenges within the field.

DPSCs are generally classified by their expression of a panel of markers, such as CD73, CD90, CD 44, and CD105, while being negative for CD34, CD45, and CD 14, among others [11,12]. Within this population of cells, various subtypes of DPSCs likely exist [4], making targeted isolation for any specific application difficult. It has been demonstrated that the specific procedures for isolating DPSCs may impact their heterogeneity [13]. The two commonly utilized approaches include digesting pulp tissue into a single-cell suspension, from which DPSCs are isolated, and the explant outgrowth method, by which intact pulp tissues are plated and DPSCs exit the tissue, adhering to the plastic. Raoof et al. and Bronckaers et al. showed that, while isolating DPSCs through enzymatic digestion yielded a substantial number of cells at a low passage rate, using tissue explants allowed for the isolation of a more homogeneous cell population [5,14]. Moreover, Raoof et al. and Huang et al. revealed that cells isolated through enzyme digestion exhibited a higher rate of proliferation compared to those isolated using the explant outgrowth method [3,5].

One approach that may be effective in initially isolating cells from digested pulp tissues is selection via differential adhesion to fibronectin. Fibronectin is a key extracellular matrix protein that can impact cell behavior and phenotype, given the interaction between fibronectin and alpha-5/beta-integrin receptors on the cell surface [15]. This interaction activates the intracellular mechanisms involved in odontoblast differentiation and the initiation of reparative dentinogenesis [16,17]. Earlier studies have employed this technique to isolate multipotent progenitors from both skin and bone marrow [18,19]. Similarly, articular-cartilage-derived progenitor cells isolated through fibronectin adhesion expressed MSC surface markers exhibited increased telomerase activity, possessed a high capacity for proliferation, and demonstrated the ability to differentiate down multiple connective tissue lineages [20,21]. In terms of DPSC isolation, initial fibronectin selection has been shown to induce the expression of mineralizing phenotype markers [22,23] and to increase their rate of proliferation [2,24]. While fibronectin has been used in studies to enhance DPSC adhesion to biomaterial scaffolds [25], it has not been fully evaluated in terms of its ability to differentially isolate distinct populations of DPSCs from human pulp tissue. Given these previous findings, it is possible that the fibronectin selection of cells from pulp tissues will generate DPSCs that proliferate rapidly while retaining their fundamental characteristics as progenitor cells. Direct comparisons, however, relating to the specific properties of fibronectin-selected cells vs. other subpopulations in the pulp are somewhat lacking. While there are many methods to isolate DPSCs, exploiting the relationship between fibronectin adhesion and retaining their fundamental properties may be a means to broadly select a population of cells from dental pulp that could be rapidly expanded while retaining their multipotency for use in various translational applications.

Therefore, this study aims to isolate and analyze DPSC proliferative capacity, cell surface marker expression, and the osteogenic potential of DPSCs originating from three different isolated populations: fibronectin-adherent (FA) and non-fibronectin-adherent (NFA) cells from single-cell suspensions, along with cells derived from whole pulp explants.

## 2. Materials and Methods

### 2.1. Isolation of Human Dental Pulp Cells

**Figure 1:**
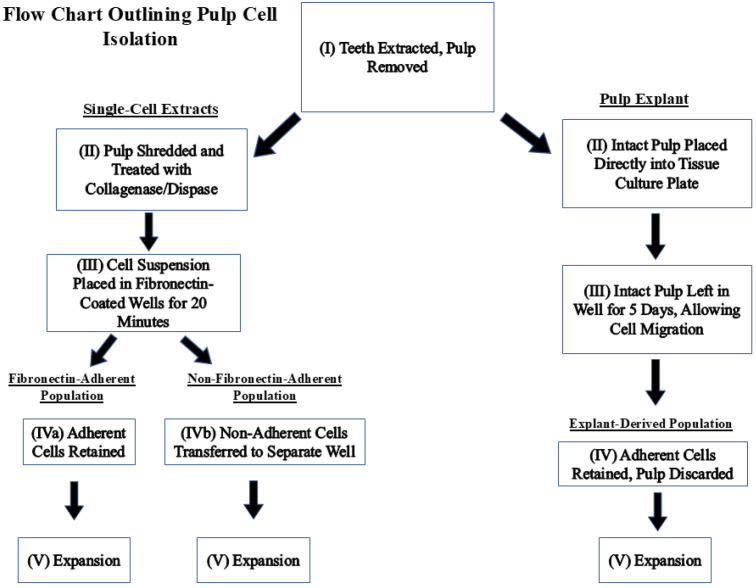
Flow chart describing how fibronectin-adherent, non-fibronectin-adherent, and pulp-explant-derived populations were obtained.

Dental pulp tissues were harvested from non-carious permanent molars and premolars extracted at the Dental clinic of the University of Nevada Las Vegas, School of Dental Medicine. The study proposal was approved by the UNLV Institutional Review Board (IRB number UNLV-2022-28, most recent exemption renewal 7 August 2024) before data collection and tissue harvesting. Figure 1 is a flow chart outlining the steps taken to isolate each population.

The extracted teeth were placed into α-MEM media (Gibco™ Modified Essential Medium α (1×) + GlutaMAX ™ with 10% *v*/*v* FBS, 1% L-ascorbic acid, 1% *v*/*v* penicillin/streptomycin, Thermo Fisher Scientific, Waltham, MA, USA) and stored no more than 1 h prior to pulp extraction. To expose the pulp chamber, 2 mm grooves were made along the cement–enamel junction using a (Dremel^®^) rotary tool (Bosch Tool Corporation, Farmington Hills, MI, USA)while ensuring there was abundant irrigation with a saline solution, facilitating the subsequent sectioning process. Using this groove, the teeth were cracked open using a wafer tweezer, exposing the pulp chambers. Pulp tissues were collected using forceps and NiTi Endo Hand K-Files (Dentsply Sirona, Charlotte, NC, USA) from the pulp canals and combined into the media.

To isolate FA and NFA populations, the pulps were finely shredded using a scalpel, treated with pre-warmed 4 mg/mL collagenase/dispase (Sigma-Aldrich, Saint Louis, MO, USA), and incubated at 37 °C, 5% CO_2_, for 1 h. The digested tissues were filtered through a 70 µM cell strainer (Thermo Fisher Scientific, Waltham, MA, USA) and washed with media. The filtered cells were centrifuged at 400 rpm for 5 min and washed twice in culture media to ensure complete removal of collagenase/dispase. The populations were generated as described in Section 2.2. For the explant culture, the intact pulp was removed from a bisected molar and placed directly in a plate well with α-MEM media for 5 days then removed. The remaining adherent cells were retained in the well, serving as the ‘explant-derived’ culture.

### 2.2. Fibronectin Selection

Fibronectin, derived from human plasma at a concentration of 10 µg/mL (Sigma), was reconstituted in 0.1 M PBS+ (PBS containing 1 mM Ca^2+^ and 1 mM Mg^2+^, pH 7.4) (Sigma). This suspension was used to coat the base of 10 cm^2^ 6-well plates (Greiner Bio-One, Monroe, NC, USA) with 1 mL/well, which were kept at 4 °C overnight. The plates were aspirated before adding cells. A single-cell suspension, resuspended in 1 mL serum-free medium, was seeded into the precoated wells at a density of 4 × 10^3^ cells/cm^2^ for 20 min at 37 °C in a 5% CO_2_ incubator. (Thermo Fisher Scientific, Waltham, MA, USA) Adherent cells were retained in the wells until confluent, constituting the FA populations. Any non-adherent cells were collected and seeded into separate uncoated tissue culture plastic wells. The adherent cells from this population served as the NFA isolates used in the subsequent experiments.

### 2.3. Sub-Culturing of DPSCs and Calculating Cumulative Population Doublings

When the DPSCs reached 80% confluence, sub-culturing was initiated. The cells were washed with 10 mL of sterile PBS and detached by incubating with 3 mL of StemPro^®^ Accutase (STEMCELL Technologies, Vancouver, BC, Canada) for 5 min. Quadruplicate cell counts were performed using a hemocytometer and then averaged. From counts at each passage over the entire culture period, the population doubling rate (PD) was calculated, allowing for a comparison of growth rates between the three isolates over time [24,26].

### 2.4. Flow Cytometric Surface Marker Expression Analysis

Cells from the 10th passage were utilized in all experiments in order to consistently ensure sufficient cell numbers across all three populations and also to monitor cell characteristics towards the end of their expansion continuum, as might be necessary when using pulp isolates for larger-scale tissue engineering applications. DPSC marker expression was analyzed using a BD Stemflow™ Human MSC Analysis kit (BD Biosciences, San Jose, CA, USA) by flow cytometry. This kit contains antibodies to common MSC markers CD73, CD90, CD105, and CD 44 and non-MSC markers CD34, CD45, CD11b, CD19, and HLA-DR, along with appropriate negative controls.

**Figure A1:**
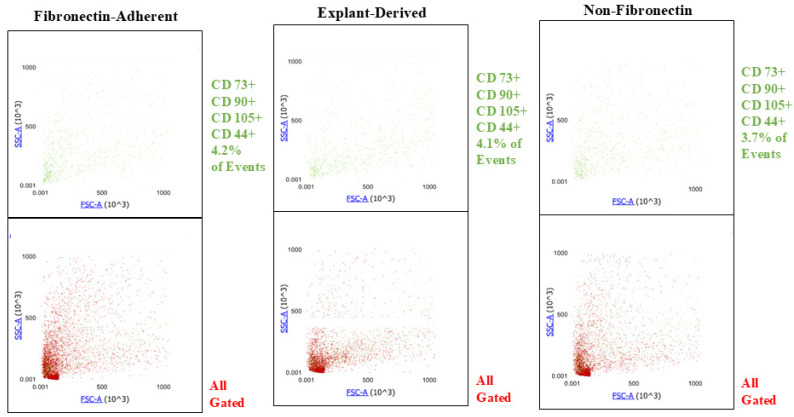
Plots showing the SSC *y* axis) and FSC (*x* axis profiles of events positive for all 4 markers (green) and their distribution within the entire gated population of events (red) for all three isolated populations. The proportions of quadruplicate positive events are similar between all three populations, and there is no apparent difference in their distribution across the gated population.

**Figure A2:**
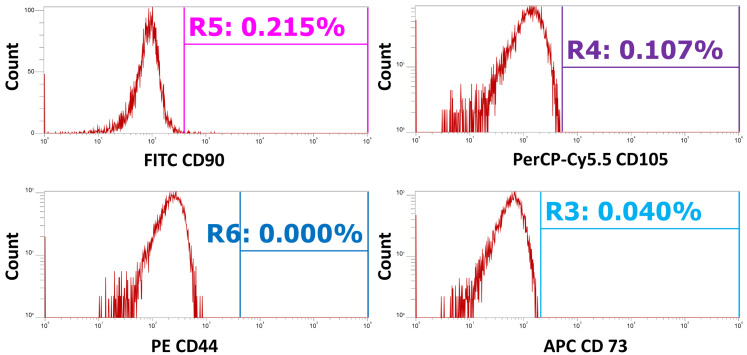
Unstained controls were used to set the positive gating for all markers. This figure shows the unstained controls plotted as event count (*x* axis) versus the relative signal intensity for each channel (*y* axis). Gates are placed based on the distribution of the unstained cells to show the threshold as to what are considered ‘positive’ events.

Cells from all three cultures were harvested by treatment with StemPro^®^ Accutase^®^ (STEMCELL Technologies, Vancouver, BC, Canada) and placed in sample tubes prepared following the manufacturer’s instructions. Both the positive and negative control tubes were incubated in the dark for 30 min at room temperature. The cells were washed twice with and resuspended in 500 µL of BD Pharmingen™ Stain Buffer (BD Biosciences, San Jose, CA, USA) and analyzed using an Attune™ NxT flow cytometer (Thermo Fisher Scientific, Waltham, MA, USA), with 20,000 events collected for each sample. Compensation settings were set using UltraComp eBeads™ compensation beads (Invitrogen, Carlsbad, CA, USA) and fluorophore-conjugated antibodies from the kit. Gating was performed on forward scatter (FSC) and side scatter (SSC) to exclude debris and subsequently gated on a Forward Scatter Height (FSH-H) vs. Forward Scatter Width (FSC-W) plot to remove potential doublets. Gates to determine positive events for all markers were set using unstained cells and isotype controls provided in the kit. Gated cells positive for all four markers and their distribution in the whole gated population are given in Figure A1 (Appendix A). A representative example of negative control gating is given in Figure A2 (Appendix A).

### 2.5. Incubation in Inductive Media

Cells from the 10th passage of all three isolates were plated at a density of 5 × 10^4^ cells/cm^2^. Each of the isolates was cultured in triplicate in either standard culture media, to serve as controls, or incubated in MSC Osteogenic Differentiation Medium (PromoCell GmbH, Heidelberg, Germany) for 7 days.

### 2.6. RNA Isolation

Total RNA was extracted from the cells cultured in osteogenic differentiation media and α-MEM media (negative control) by using an RNeasy^®^ mini-prep kit (Qiagen, Germantown, MD, USA) following the manufacturer’s guidelines. The concentration and purity were determined using a NanoDrop™ One Spectrophotometer (Thermo Fisher Scientific, Waltham, MA, USA), and purity was calculated from the A260/280 ratio.

### 2.7. Reverse Transcription-PCR (RT-PCR) for cDNA Synthesis

The cDNA synthesis process utilized a High-capacity cDNA Reverse Transcription Kit (Thermo Fisher Scientific, Waltham, MA, USA). Total RNA concentration was adjusted to 0.5 µg in nuclease-free water and combined with reverse transcription master mix with random primers at a 1:1 ratio for a total volume of 60 µL. RT-PCR was performed by a GeneAmp PCR System 9700 Thermal Cycler (Thermo Fisher Scientific, Waltham, MA, USA) under the following conditions: primers annealed at 25 °C for 10 min, RNA reverse-transcribed at 37 °C for 120 min, and enzyme-inactivated at 85 °C for 5 min.

### 2.8. Gene Expression Analysis via Real-Time Quantitation PCR (qPCR)

The relative quantitation of osteogenic gene expression was performed by qPCR using 50 ng of cDNA template, a TaqMan^®^ Fast Advanced Master Mix (Thermo Fisher Scientific, Waltham, MA, USA), and a TaqMan^®^ Gene Expression assay (Thermo Fisher Scientific, Waltham, MA, USA) (Table 1) on a QuantStudio™ 3 Real-Time PCR system (Thermo Fisher Scientific, Waltham, MA, USA). Briefly, gene expression was examined for the osteogenic markers BGLAP, the gene that encodes osteocalcin, SPP1, the gene that encodes osteopontin, and RUNX2, a gene that encodes Runt-related transcription factor 2. RPLP0, the gene that encodes 60S acidic ribosomal protein PO, was used as a housekeeping reference gene. The qPCR instrument was set up in the fast-cycling mode with the following parameters: polymerase enzyme activation at 95 °C for 20 s followed by 40 cycles of denaturation at 95 °C for 1 s and annealing-extension at 60 °C for 20 s. Each experiment was conducted in triplicate and run with a negative control. The comparative Ct method was used to analyze mRNA expression levels normalized with the reference gene.

### 2.9. Statistical Analysis

The cumulative population doubling data for all three DPSC isolates were compared using a repeated measures ANOVA analysis, with statistical significance considered to be *p* < 0.05. Statistical tests were performed using SPSS software (Version 28.0.1.0 (142) IBM, Armonk, NY, USA). For the q-PCR analyses, all assays were conducted in three independent experiments, i.e., separate cell cultures and RNA extractions (*n* = 3), with three subsequent experimental replicates for each experiment. The Mann–Whitney U Test analysis was employed to assess the significance of relative expression between the untreated control group and the treated group, with significance assumed at *p* < 0.05 (Excel 2016). The standard error of the mean (SEM) of the RQ was calculated. An SEM approaching zero suggests that the estimated value is nearly identical to the true value.

## 3. Results

### 3.1. Cumulative Population Doublings

**Figure 2:**
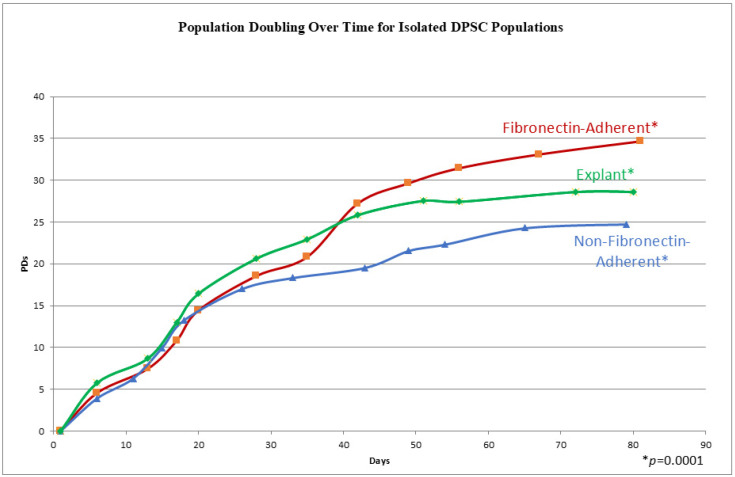
A graph showing the cumulative population doublings (PDs) for isolates at each passage on the *y* axis, with the days in culture on the *x* axis. Each data point represents a passage and a quadruplicate count. A repeated measures ANOVA shows a *p*-value of 0.0001, indicating significantly different cumulative population doublings over time.

The cells from all three populations had a similar morphology throughout the whole culture period, which consisted of a stellate, fibroblast-like appearance typical of DPSCs [27], and no differences could be distinguished between the populations by direct examination. In order to track the proliferation kinetics of each of these populations, their population doubling rates, i.e., the cumulative number of times a cell population doubles during cell culture, were measured over time. The three experimental groups completed a total of 11–13 passages within 72–81 days. In Figure 2, the three experimental groups illustrate a similar sigmoidal growth pattern, characterized by an increase in cell culture at the initial stages, followed by steady population growth over time. The explant-derived cultures showed the highest population doublings from passages 0 to 6. After passage 6 (beyond 40 days), the FA isolate surpassed the growth of the explant cells, consistently maintaining the highest population throughout the subsequent days. The FA DPSCs consistently had higher population doublings than the NFA isolate. Additionally, the explant-derived cell doubling rate began to plateau at passage 8, a potential indicator of senescence, which was earlier than the other two groups. The FA and NFA populations were still expanding at 73 days post-isolation. The examination of cumulative PD numbers through repeated measures ANOVA identified significant differences in the cumulative population doublings between groups, as demonstrated by a *p*-value of 0.0001.

### 3.2. Immunophenotypic Characterization of Dental Pulp Stem Cells

**Figure 3:**
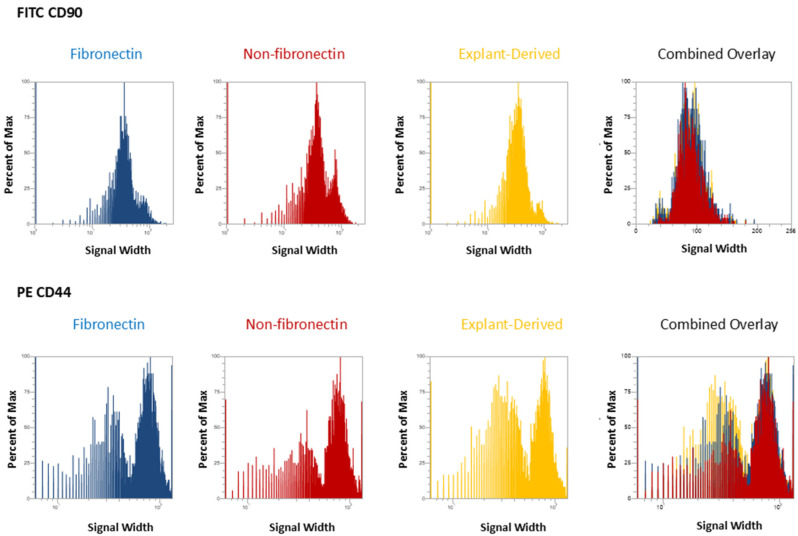
Flow cytometric analysis of mesenchymal cell surface marker expression on the three populations isolated from 20,000 total events. The graphs show the distribution of events positive for CD 90 labeled with FITC and CD 44, labeled with PE, plotted with the percent of the maximum signal intensity (*y* axes) against the event signal width (*x* axes).

**Figure 4:**
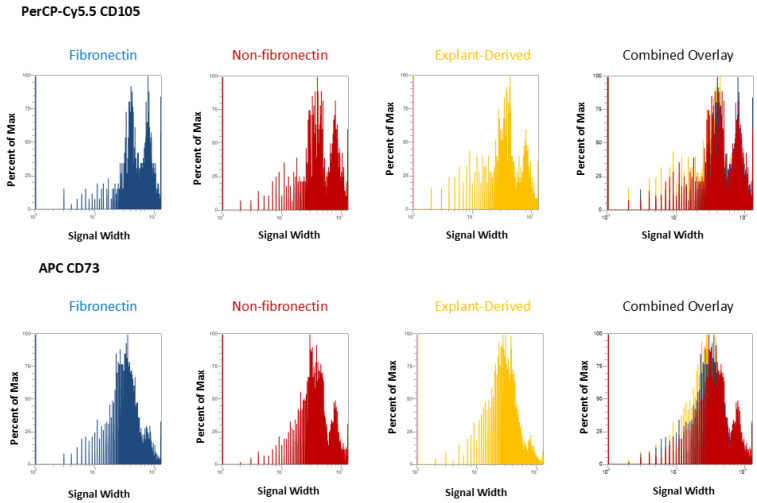
Flow cytometric analysis of mesenchymal cell surface marker expression on the three populations isolated from 20,000 total events. The graphs show the distribution of events positive for CD105, labeled with PerCP-Cy5.5 and CD73 labeled with APC for each population, plotted with the percent of the maximum signal intensity (*y* axes) against the event signal width (*x* axes).

**Figure 5:**
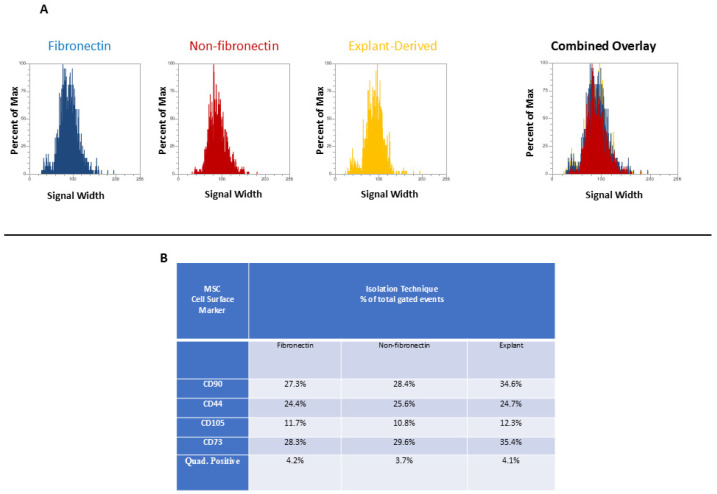
(**A** and **B**) (**A**) The distribution of quadruplicate (CD90, CD44, CD 105, and CD 73)-positive events (*y* axis) versus side scatter (*x* axis). (**B**) A table listing the percentage of total events positive for each individual marker as well as the percentage of total events positive for all four markers.

In Figure 3 and Figure 4, the distribution of positive events for all surface markers is shown. Figure 3 shows the distribution of events positive for CD 90 andCD44, between the three isolated populations when plotted in terms of the percentage of the total number of events (*y* axes) versus relative signal intensity (*x* axes). Figure 4 shows the same for CD105 and CD73. In both figures, the combined overlay for each individual marker shows that these profiles are highly similar between the three isolated populations of DPSCs. Similarly, Figure 5A shows that events positive for all four surface markers have a similar distribution between the three isolated populations, indicating that ‘quadruplicate’-positive cells are similar in terms of number and distribution within all three populations. Figure 5B gives the percentage of total positive events for each marker across the three isolated populations, in addition to the percentage of events in each population that are positive for all four markers in a table. The proportion of cells positive for any given marker is similar between each isolated population, as is the proportion of cells positive for all four markers.

### 3.3. mRNA Expression of Osteodifferentiation Genes

#### 3.3.1. RUNX2

**Figure 6:**
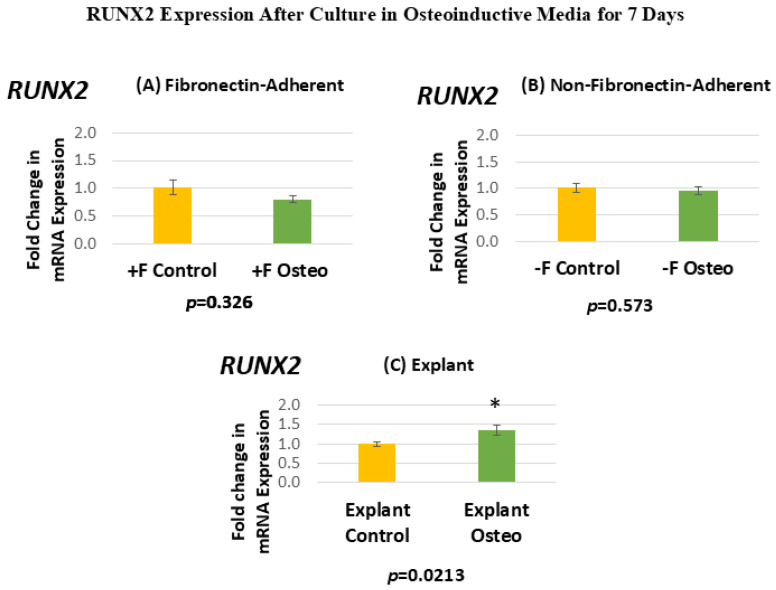
(**A**–**C**) Mean fold changes, or relative quantification (RQ), for the expression of RUNX2 following a 7-day exposure to osteogenic differentiation media in the fibronectin-adherent (**A**), non-fibronectin-adherent (**B**), and explant-derived (**C**) populations. Fold changes are compared to controls cultured for 7 days in normal culture media. Statistically significant differences are indicated by * (*p* < 0.05).

Figure 6 shows the RUNX2 expression for each isolated population after 7 days in the osteoinductive media compared against the untreated controls. Figure 6A, for the FA isolates, shows that RUNX2 gene expression has a mean fold change of 0.81 in the osteogenic-media-treated group compared to the untreated controls. Figure 6B, for the NFA isolates, shows that RUNX2 gene expression has a mean fold change of 0.95 in the osteogenic-media-treated group compared to the untreated controls. Figure 6C, for the explant-derived isolates, shows that RUNX2 gene expression has a mean fold change of 1.35 in the osteogenic-media-treated group compared to the untreated controls.

Neither the FA nor the NFA DPSC isolate displays a significant increase in the level of RUNX2 expression after culture in the osteogenic media compared to the untreated controls. In contrast, the osteogenic-media-treated explant-derived DPSC isolate demonstrates a significant increase of around 35% in RUNX2 mRNA. The standard error of the mean for all groups ranges from 0.06 to 0.13, indicating that the calculated RQs closely align with the true value.

#### 3.3.2. SPP1

**Figure 7:**
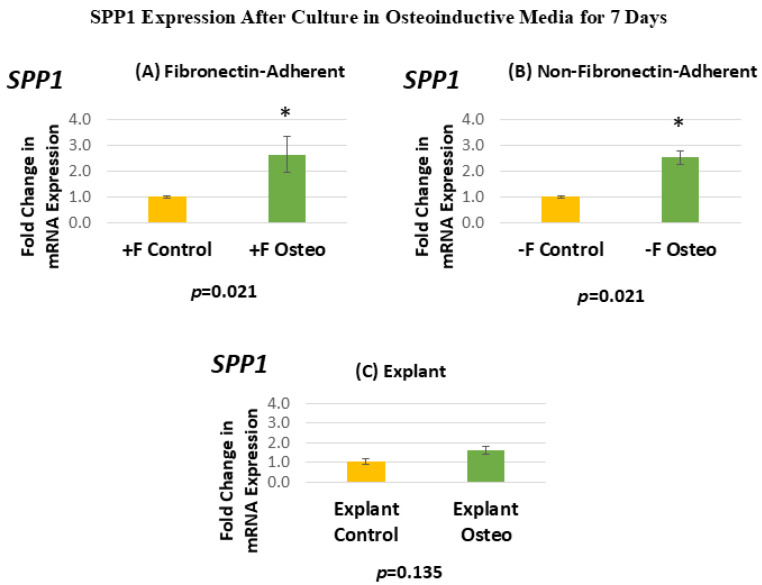
(**A**–**C**) Mean fold changes, or relative quantification (RQ), for the expression of SPP1 following a 7-day exposure to osteogenic differentiation media. Fold changes are compared to controls cultured for 7 days in normal culture media. Statistically significant differences are indicated by * (*p* < 0.05).

Figure 7 shows the SPP1 expression for each isolated population after 7 days in the osteoinductive media compared against the untreated controls. Figure 7A, for the FA isolates, shows that SPP1 gene expression has a mean fold change of 2.64 in the osteogenic-media-treated group compared to the untreated controls. Figure 7B, for the NFA isolates, shows that SPP1 gene expression has a mean fold change of 2.52 in the osteogenic-media-treated group compared to the untreated controls. Figure 7C, for the explant-derived isolates, shows that SPP1 gene expression has a mean fold change of 1.6 in the osteogenic-media-treated group compared to the untreated controls.

The FA DPSC isolate displays a significant 160% increase in SPP1 mRNA expression after treatment in the osteogenic media, giving a *p*-value of 0.0213. Similarly, the NFA DPSC isolate displays a similar significant increase in SPP1, with mRNA expression in the osteogenic media treatment group increasing by 150%. In contrast, the osteogenic-media-exposed explant-derived isolates do not demonstrate a significant change in the level of SPP1. The standard error of the mean for all groups ranged from 0.05 to 0.71, indicating that the calculated RQs closely align with the true values.

#### 3.3.3. BGLAP

**Figure 8:**
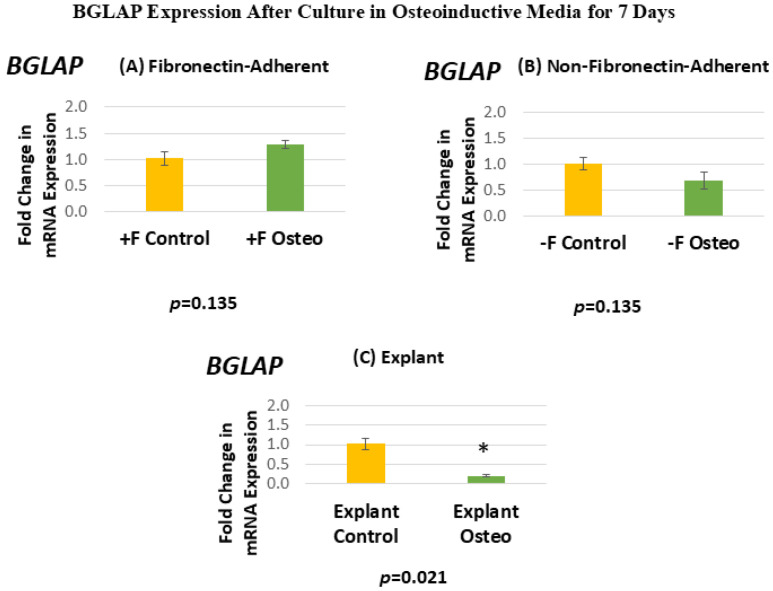
(**A**–**C**) Mean fold changes, or relative quantification (RQ), for the expression of *BGLAP* following a 7-day exposure to osteogenic differentiation media. Fold changes are compared to controls cultured for 7 days in normal culture media. Statistically significant differences are indicated by * (*p* < 0.05).

Figure 8 shows the BGLAP expression for each isolated population after 7 days in the osteoinductive media compared against the untreated controls. Figure 8A, for the FA isolates, shows that BGLAP gene expression has a mean fold change of 1.28 in the osteogenic-media-treated group compared to the untreated controls. Figure 8B, for the NFA isolates, shows that BGLAP gene expression has a mean fold change of 0.69 in the osteogenic-media-treated group compared to the untreated controls. Figure 8C, for the explant-derived isolates, shows that BGLAP gene expression has a mean fold change of 0.2 in the osteogenic-media-treated group compared to the untreated controls.

Neither the FA nor the NFA isolate displays a significant increase in the level of BGLAP expression after culture in the osteogenic media. Notably, the osteogenic-media-treated explant-derived isolate demonstrates a significant decrease in the expression of BGLAP, with expression levels falling by around 80%. The standard error of the mean for all groups ranged from 0.03 to 0.16, indicating that the calculated RQs closely align with the true values.

## 4. Discussion

There remains a significant amount of interest in the utilization of DPSCs in regenerative dentistry due to their ability to differentiate into various types of connective tissue cells, including endothelial cells, fibroblasts, and odontoblast-like cells. For example, if isolated DPSCs could be applied to diseased tissues in sufficient numbers and induced to differentiate appropriately, they could provide a means to repair damage to the dentin–pulp complex and to reduce pulpal inflammation, given their potential anti-inflammatory properties [28]. There are also many potential applications that use DPSCs to regrow and repair oral soft tissues. A significant barrier to utilizing these cells in tissue engineering applications, however, is their relative rarity within the pulp and the difficulty in isolating them, as they do not share a single easily identifiable surface marker [1,2,3,4]. Therefore, the first critical step in utilizing DPSCs therapeutically is isolating an enriched population that can be expanded significantly in vitro in a way that does not interfere with their properties as multipotent cells. Here, we examined how various isolation techniques, including fibronectin as a matrix to enrich extracted DPSCs, affect their ability to proliferate in vitro, maintain specific DPSC surface markers, and differentiate into a mineralizing cell phenotype, which are significant considerations when expanding cells for use in therapeutic applications. These data have important implications for many potential isolation techniques; indeed, these findings suggest that even if a population of pulp-derived cells was sorted based on a panel of commonly agreed DPPC surface markers, without initial enrichment through the preferential adhesion to fibronectin, one might expect to find subpopulations that have significantly different growth kinetics or differentiation potential [4,25].

In this study, a notable difference in population doublings between the dental pulp stem cells isolated through fibronectin selection and the other isolates was observed. This suggests that fibronectin isolation contributes to improved population growth, as evidenced by the significantly higher cumulative population doubling over time. By post-isolation passage 7 (day 33), the fibronectin-adherent cells began to show a much greater rate of population doubling compared to the non-fibronectin-adherent cells and were still expanding by the end of the culture period (passage 12, day 80). At this point, the cells from the non-fibronectin-adherent cells had reached a plateau phase. These findings align with previous studies indicating that human MSCs isolated through fibronectin adhesion exhibit enhanced telomerase activity and possess a robust proliferation capacity [21,22,23]. The greater expansion potential of cells isolated by fibronectin adhesion is a crucial consideration for harvesting ample quantities of human DPSC subpopulations and assessing and developing them for clinical use. Moreover, the enhanced proliferative potential of fibronectin-adherent MSCs may be valuable in tissue engineering applications; for example, in the use of chitosan tissue engineering scaffolds, fibronectin has been found to be an essential component for DPSC attachment and proliferation [4,25].

With regard to the explant-derived isolates, our results are similar to previous studies by Huang et al. [2] and Raoof et al. [5], which suggested that cells obtained through enzymatic digestion exhibited a generally higher proliferation rate compared to those isolated via explant outgrowth. While the explant-derived cells demonstrated an increased PD rate at early passages, by passage 7 (day 35), they were overtaken by the single-cell fibronectin-adherent isolates. The explant-derived isolate reached a plateau phase by passage 9 (day 50) and appeared to be senescent by passage 11 (day 73), which is in contrast to the actively dividing FA and NFA isolates. While it is difficult to speculate on the mechanism behind these observations, fibronectin selection, based on a higher proportional cell surface expression of alpha-5/beta1 integrin, seems to have served to enrich a population containing more DPSCs with greater proliferation kinetics than the unenriched NFA or explant-derived DPSCs. This observation is broadly in agreement with findings that even in clonally isolated lines of DPSCs, there is significant variance in proliferation rates and time to senescence [26]. Presumably, the less enriched populations, i.e., the NFA and explant-derived DPSCs, had a greater proportion of slower-dividing cells.

As might be expected from the relative rarity of DPSCs, flow cytometry revealed that all isolates displayed relatively low proportions of cells positive for all four markers. Interestingly, this proportion was comparable across all isolates. Therefore, it does not seem that fibronectin significantly enhanced the proportion of cells positive for these four MSC cell surface markers. Similarly, the proportion of cells positive for any single MSC cell surface marker was comparable across all populations, with the proportions between the fibronectin and non-fibronectin isolates being under a 1% difference from each other. It is noteworthy that some marker expression proportions were slightly elevated in the explant-derived isolates, with the greatest difference being around a 7% higher expression in CD90 and CD73 between the explant-derived DPSCs and the other two groups. It is difficult, however, to comment on any sort of biological significance related to this relatively small observed variation based on marker expression alone, given the diversity of cellular functions to which these markers relate. Indeed, the proportion of cells positive for all four markers, which would, in theory, best describe a ‘true’ DPSC, was almost identical between the three populations and, as might be expected, small within the greater pool of isolated cells positive for at least one of the DPSC marker panel.

*RUNX2*, *SPP1*, and *BGLAP* were chosen as osteogenic markers in this study as they give a general continuity in the differentiation timeline of mineralizing-type cells. *RUNX2* is a key osteogenic transcription factor and serves as an early osteogenic differentiation marker [29]. Osteopontin (OPN), the product of the *SPP1* gene, is recognized as an early marker of mineralization pathways, while osteocalcin (OCN), the *BGLAP* gene product, is acknowledged as a later marker in the osteogenic process [30,31]. Specifically, a study by Zohar et al. [30] demonstrated that OPN is associated with cell migration and is expressed earlier in osteo-differentiation. In vivo, during mandibular bone healing in rats, it was also demonstrated that OPN is expressed early in healing, subsequently diminishing as bone healing progresses and OCN expression increases [32]. In summary, RUNX2 is an early indicator of mineralizing cell differentiation, OPN is an early protein expressed in the development of mineralizing cells, often indicating pre-osteoblast migration and attachment, and OCN is a later-stage protein, indicating the commencement of tissue matrix synthesis.

While *RUNX2* was expressed in all cultures, the osteogenic media treatment did not appear to alter its expression between the FA or NFA cells, while the explant-derived cells showed a significant increase in *RUNX2* mRNA expression. This is perhaps an indication of the baseline expression in the single-cell isolates, which was not substantially altered by the osteoinductive media, and could be due to some underlying difference in the enzymatic digestion of pulp tissues. If the cells from the digested pulp isolates, by the later stage in their expansion at which they were examined, had partially begun to differentiate down the osteogenic lineage, these results would make a certain amount of sense, given the very early expression of RUNX2 in the differentiation continuity.

Both the FA and NFA populations displayed elevated *SPP1* mRNA expression yet showed no significant difference in *BGLAP* mRNA expression after 7 days of osteogenic differentiation media treatment compared to the untreated controls. Given the fact that OPN is an early-stage marker of mineralizing cell type differentiation, it is, therefore, possible that these isolates were in the early stages of differentiation at the 7-day treatment mark and, therefore, had an elevated expression of *SPP1*. In contrast, they might have not yet increased their expression of *BGLAP*, as OCN is generally a much later marker in the process of osteodifferentiation. Both isolates showed a similar increase in expression when exposed to the osteoinductive media, indicating that they were at a similar point in the continuity of osteoblast differentiation and thus had a similar capability for osteodifferentiation. It is possible that longer culture times in osteogenic media would have resulted in increased *BGLAP* expression in both of these isolates.

In contrast, the explant-derived cells showed a significant decrease in *BGLAP* expression following 7 days in the osteoinductive media compared to the untreated controls. The reason for this is somewhat unclear, especially taken in conjunction with the increase in *RUNX2* expression seen in the treated explant-derived isolate. This could indicate that the explant-derived cells were at an earlier time point in the continuum of osteodifferentiation, as *RUNX2* is an upstream regulator of this process, meaning that the treated cells were at an earlier stage of this process than the FA or NFA cells, given their *SPP1* expression, which controls the downstream product OPN. This less rapid onset of differentiation might be explained by the fact that the explant-derived cells had a considerably decreased rate of population doubling by the 10th passage. It is possible that these cells, at this later passage, had lost some capacity for downstream osteogenic marker expression (*BGLAP* or *SPP1*), despite the slightly increased expression of the more upstream regulator *RUNX2*, and, therefore, did not progress as far down this pathway as the other populations. It is also possible that the explant-derived cells were less efficient at osteo-differentiation and were unable to progress down the osteoblast lineage as rapidly as their FA or NFA counterparts and were simply in an earlier phase of differentiation at the end of the culture period in the osteogenic media.

All three isolated populations demonstrated a retained capacity for osteodifferentiation to some extent after extended expansion in vitro. This is a critical consideration for any DPSC isolate, as many translational applications in regenerative dentistry pertain to inducing mineralizing cell type differentiation and function. The single-cell isolates, however, appeared to advance further down the mineralizing cell phenotype lineage compared to the explant-derived cells, given their greater expression of later-stage osteodifferentiation markers after 7 days. Further work is clearly needed to explore if this is a result of extended replication itself or a direct result of the specific isolation technique employed.

A limitation of this study is that all cells studied are from a single donor, which represents a significant difficulty in terms of clinical translation and substantially limits the breadth of applicability for the results presented. One might expect donor variability in terms of the properties examined here, depending on a large number of factors (age, specific tooth extracted, genetic background, general health condition). These experiments would need to be carried out on a relatively broad selection of donors, preferably age-range-matched, in order to establish a better understanding of different isolation techniques yielding populations with different properties, which represents the clear future direction of this work.

## 5. Conclusions

Based on these data, it appears that initial fibronectin selection of single-0cell isolates from digested pulp appears to yield a population of cells with a much higher proliferation rate over longer-term culture compared to single-cell isolates that were not initially fibronectin-adherent or whole-pulp-explant-derived cells. Further, DPPCs from single-cell isolates potentially demonstrated a greater capacity for osteodifferentiation than whole-pulp-explant-derived cells. Our findings, therefore, support the use of fibronectin as a potentially valuable selection tool capable of promoting cellular expansion without the loss of specific surface markers, both of which are key considerations in potential therapeutic applications of isolated DPSCs.

## Figures and Tables

**Table 1:** List of off-the-shelf TaqMan^®^ Gene Expression Assays containing primers and minor groove binder (MGB) probe.

Genes	Reporter Dye	Assay ID
BGLAP	FAM	Hs01587814_g1
SPP1	FAM	Hs00959010_m1
RUNX2	FAM	Hs01047973_m1
RPLP0	VIC	Hs00420895_gH

## Data Availability

The raw data supporting the conclusions of this article will be made available by the authors upon request.

## References

[1] Xuan K., Li B., Guo H., Sun W., Kou X., He X., Zhang Y., Sun J., Liu A., Liao L. (2018). Deciduous autologous tooth stem cells regenerate dental pulp after implantation into injured teeth. Sci. Transl. Med..

[2] Huang G.T.-J., Gronthos S., Shi S. (2009). Mesenchymal stem cells derived from dental tissues vs. those from other sources: Their biology and role in regenerative medicine. J. Dent. Res..

[3] Huang G.T.-J., Sonoyama W., Chen J., Park S.H. (2006). In vitro characterization of human dental pulp cells: Various isolation methods and culturing environments. Cell Tissue Res..

[4] Kok Z.Y., Alaidaroos N.Y.A., Alraies A., Colombo J.S., Davies L.C., Waddington R.J., Sloan A.J., Moseley R. (2022). Dental pulp stem cell heterogeneity: Finding superior quality “Needles” in a dental pulpal “Haystack” for regenerative medicine-based applications. Stem Cells Int..

[5] Raoof M., Yaghoobi M.M., Derakhshani A., Kamal-Abadi A.M., Ebrahimi B., Abbasnejad M., Shokouhinejad N. (2014). A modified efficient method for dental pulp stem cell isolation. Dent. Res. J..

[6] Harrington J., Sloan A.J., Waddington R.J. (2014). Quantification of clonal heterogeneity of mesenchymal progenitor cells in dental pulp and bone marrow. Connect. Tissue Res..

[7] Zhao Y., Zheng Y., Eichhorn W., Klatt J., Henningsen A., Kluwe L., Friedrich R.E., Gosau M., Smeets R. (2019). Enriching Stem/Progenitor Cells from Dental Pulp Cells by Low-density Culturing. In Vivo.

[8] Di T., Wang L., Cheng B., Guo M., Feng C., Wu Z., Wang L., Chen Y. (2024). Single-cell RNA sequencing reveals vascularization-associated cell subpopulations in dental pulp: PDGFRβ+ DPSCs with activated PI3K/AKT pathway. Stem Cells.

[9] Alraies A., Canetta E., Waddington R.J., Moseley R., Sloan A.J. (2019). Discrimination of Dental Pulp Stem Cell Regenerative Heterogeneity by Single-Cell Raman Spectroscopy. Tissue Eng. Part C Methods.

[10] Patil V.R., Kharat A.H., Kulkarni D.G., Kheur S.M., Bhonde R.R. (2018). Long term explant culture for harvesting homogeneous population of human dental pulp stem cells. Cell Biol. Int..

[11] Yamada Y., Nakamura S., Ito K., Sugito T., Yoshimi R., Nagasaka T., Ueda M. (2010). A feasibility of useful cell-based therapy by bone regeneration with deciduous tooth stem cells, dental pulp stem cells, or bone-marrow-derived mesenchymal stem cells for clinical study using tissue engineering technology. Tissue Eng. Part A.

[12] Al Madhoun A., Sindhu S., Haddad D., Atari M., Ahmad R., Al-Mulla F. (2021). Dental pulp stem cells derived from adult human third molar tooth: A brief review. Front. Cell Dev. Biol..

[13] Rodas-Junco B.A., Villicaña C. (2017). Dental pulp stem cells: Current advances in isolation, expansion and preservation. Tissue Eng. Regen. Med..

[14] Bronckaers A., Hilkens P., Fanton Y., Struys T., Gervois P., Politis C., Martens W., Lambrichts I. (2013). Angiogenic properties of human dental pulp stem cells. PLoS ONE.

[15] Zhu Q., Safavi K.E., Spangberg L.S. (1998). The role of integrin β1 in human dental pulp cell adhesion on laminin and fibronectin. Oral Surg. Oral Med. Oral Pathol. Oral Radiol. Endod..

[16] Yoshiba K., Yoshiba N., Nakamura H., Iwaku M., Ozawa H. (1996). Immunolocalization of fibronectin during reparative dentinogenesis in human teeth after pulp capping with calcium hydroxide. J. Dent. Res..

[17] Tziafas D., Panagiotakopoulos N., Komnenou A. (1995). Immunolocalization of fibronectin during the early response of dog dental pulp to demineralized dentine or calcium hydroxide-containing cement. Arch. Oral Biol..

[18] D’Ippolito G., Diabira S., Howard G.A., Menei P., Roos B.A., Schiller P.C. (2004). Marrow-isolated adult multilineage inducible (MIAMI) cells, a unique population of postnatal young and old human cells with extensive expansion and differentiation potential. J. Cell Sci..

[19] Jones P.H., Watt F.M. (1993). Separation of human epidermal stem cells from transit amplifying cells on the basis of differences in integrin function and expression. Cell.

[20] Korpershoek J.V., Rikkers M., de Windt T.S., Tryfonidou M.A., Saris D.B.F., Vonk L.A. (2021). Selection of highly proliferative and multipotent meniscus progenitors through differential adhesion to fibronectin: A novel approach in meniscus tissue engineering. Int. J. Mol. Sci..

[21] Williams R., Khan I.M., Richardson K., Nelson L., McCarthy H.E., Analbelsi T., Singhrao S.K., Dowthwaite G.P., Jones R.E., Baird D.M. (2010). Identification and clonal characterisation of a progenitor cell sub-population in normal human articular cartilage. PLoS ONE.

[22] Lee H., Bae A., Kim J., Kingsley K. (2023). Differential Effects of Extracellular Matrix Glycoproteins Fibronectin and Laminin-5 on Dental Pulp Stem Cell Phenotypes and Responsiveness. J. Funct. Biomater..

[23] Omer A., Al-Sharabi N., Qiu Y., Xue Y., Li Y., Fujio M., Mustafa K., Xing Z. (2020). Biological responses of dental pulp cells to surfaces modified by collagen 1 and fibronectin. J. Biomed. Mater. Res..

[24] Alaidaroos N.Y.A., Alraies A., Waddington R.J., Sloan A.J., Moseley R. (2021). Differential SOD2 and GSTZ1 profiles contribute to contrasting dental pulp stem cell susceptibilities to oxidative damage and premature senescence. Stem Cell Res. Ther..

[25] Sana F.A., Yurtsever M.Ç., Bayrak G.K., Tunçay E.Ö., Kiremitçi A.S., Gümüşderelioğlu M. (2017). Spreading, proliferation and differentiation of human dental pulp stem cells on chitosan scaffolds immobilized with RGD or fibronectin. Cytotechnology.

[26] Alraies A., Alaidaroos N.Y.A., Waddington R.J., Moseley R., Sloan A.J. (2017). Variation in human dental pulp stem cell ageing profiles reflect contrasting proliferative and regenerative capabilities. BMC Cell Biol..

[27] Kang M.K., Colombo J.S., D’souza R.N., Hartgerink J.D. (2014). Sequence Effects of Self-Assembling MultiDomain Peptide Hydrogels on Encapsulated SHED Cells. Biomacromolecules.

[28] Galler K.M., Weber M., Korkmaz Y., Widbiller M., Feuerer M. (2021). Inflammatory Response Mechanisms of the Dentine–Pulp Complex and the Periapical Tissues. Int. J. Mol. Sci..

[29] Xu J., Li Z., Hou Y., Fang W. (2015). Potential mechanisms underlying the *RUNX2* induced osteogenesis of bone marrow mesenchymal stem cells. Am. J. Transl. Res..

[30] Zohar R., Cheifetz S., McCulloch C.A.G., Sodek J. (1998). Analysis of intracellular osteopontin as a marker of osteoblastic cell differentiation and mesenchymal cell migration. Eur. J. Oral Sci..

[31] Tsao Y.-T., Huang Y.-J., Wu H.-H., Liu Y.-A., Liu Y.-S., Lee O.K. (2017). Osteocalcin mediates biomineralization during osteogenic maturation in human mesenchymal stromal cells. Int. J. Mol. Sci..

[32] Colombo J.S., Balani D., Sloan A.J., Crean S.J., Okazaki J., Waddington R.J. (2011). Delayed osteoblast differentiation and altered inflammatory response around implants placed in incisor sockets of type 2 diabetic rats. Clin. Oral Implant. Res..

